# Is there sufficient evidence for the association between executive dysfunction and academic performance in adolescents with major depressive disorder?: a systematic review

**DOI:** 10.1007/s00787-023-02275-9

**Published:** 2023-08-10

**Authors:** Justyna Urbańska-Grosz, Maciej Walkiewicz, Emilia J. Sitek

**Affiliations:** 1 Rehabilitation Department of Child and Adolescent Psychiatry, Gdanskie Centrum Zdrowia, Gdansk, Poland; 2 Laboratory of Clinical Neuropsychology, Neurolinguistics and Neuropsychotherapy, Division of Neurological and Psychiatric Nursing, Faculty of Health Sciences, Medical University of Gdansk, Gdansk, Poland; 3 Division of Quality of Life Research, Department of Psychology, Faculty of Health Sciences, Medical University of Gdansk, Gdansk, Poland; 4 Department of Neurology, St. Adalbert Hospital, Copernicus PL, Gdansk, Poland

**Keywords:** Executive function, Inhibition, Depression, Adolescence, Assessment, Education

## Abstract

Adult depression, undoubtedly associated with executive dysfunction, leads to poor work performance. As depression in adolescents may have a negative impact on school performance, we aimed to analyse the possible relationship between selected executive deficits and academic performance. Executive dysfunctions may have more severe consequences on school performance at high school, as this stage of education requires engagement in long-term goals, whether writing an essay or preparing for an exam. Whilst inhibitory control is necessary at all educational stages, it seems that planning and decision-making play a greater role in high school than in primary school. We reviewed studies on executive functions conducted in adolescents diagnosed with major depressive disorder (MDD) to establish the possible relationship between executive processes and school performance in depressed adolescents. The search identified 5 studies addressing planning and decision-making in adolescents with MDD, but none of those studies reported educational achievement. We identified a considerable gap in the research on the functional impact of depression in adolescents. Identifying the link between specific executive deficits and school performance could guide tailored therapeutic interventions.

## Introduction

Depression has a negative impact on school performance in adolescents [1], which may be also persistent [2]. Whilst executive functions (EF) are crucial components of success in education, being an important area for children and adolescents, EF deficits remain also one of the main manifestations of cognitive impairment in adult depression [3]. However, data on executive dysfunction in adolescent depression are not homogenous [4]. Unlike in the adult population, the profile of EF deficits in paediatric populations with major depressive disorder (MDD) is inconsistently described in the literature and many studies yield negative results [4].

EF as a multi-dimensional construct enable us to prioritise, plan, initiate, maintain and inhibit goal-directed activity, as well as monitor our performance, coordinate the performance of multiple activities, and modify our strategy if it turns out to be ineffective or if we need to respond to changes in our environment [5]. Adolescence remains the most important developmental period as far as executive functions are concerned [6]. However, executive functions continue to evolve up to early adulthood [7]. The prefrontal cortex develops much later than other brain areas [6] and any disorder affecting the adolescent brain may disrupt the evolution of executive skills. Thus, executive deficits in depressed adolescents are likely to be the effect of interaction between the pathological and developmental processes. Of note, these developmental processes are by no means unidirectional or strictly parallel as the linear increase in frontal white matter is accompanied by the non-linear decrease in frontal grey matter density [7]. The evolution of executive skills in adolescence, especially of hot executive skills, such as affective decision-making, is unlikely to be linear [8], which adds further complexity to the study of executive dysfunction in depressed adolescents. The age-related increase in simultaneous activity of the frontal–parietal network is related to the improvement in response inhibition in several studies [9]. The relationship between executive function components and educational achievement varies across ages [10]. Whilst at the early school age, inhibitory control is of major importance, later, when basic academic skills (reading, writing, calculation) are not sufficient to succeed, e.g. when one has to write essays or study for final exams, the role of planning multi-step activity in educational attainment seems to increase considerably. Self-regulation strategies are important both in the classroom and during individual study time.

The association of EF with academic performance is well documented in the literature [11]. Currently, in the era of intensive social media use, it seems that the range of possible distractors has expanded notably, which puts an additional burden on inhibitory processes to ensure that one stays focussed on the selected goal-directed activity such as studying. Studies dating back to previous decades showed that tests of poor inhibitory control may be used as measures of distractibility [12]. Of note, the research on the association of EF and academic outcomes focussed mainly on younger children and basic academic skills, such as reading, writing or mathematics, and highlighted especially the role of working memory and inhibition [13]. Some aspects of EF seem to be related in particular to performance in some domains (e.g. association of working memory with literacy or the association of problem-solving with advanced mathematics that goes beyond calculation tasks) [10], whilst others (e.g. inhibition) contribute substantially to performance in all educational areas [14]. As poor educational attainment in adolescents may have serious future consequences in terms of further education pathways or career options and as executive functions develop in adolescence and young adulthood [15, 16], we decided to focus on the adolescent population. The complexity of educational challenges after having mastered basic academic skills (e.g. necessity to plan study time and resist social media distractors whilst studying) adds to the broader context of social development, especially creating a balance between family expectations and peer pressure and successful development of autonomy. These simultaneous factors, school, family and peer relationships lead to the necessity to prioritise some tasks over others and re-prioritise them on the ongoing basis. These competing demands of the environment require the engagement of meta-tasking, effective interleaving sub-goals of one task with sub-goals of another task [5]. Depressed individuals are supposed to present with features of dysexecutive, apathetic and inappropriate syndromes, whilst the relevance of disorganised syndrome (associated with meta-tasking) in depression is so far unclear [5].

In our review, we aim to describe possible relationships between selected aspects of executive functions (inhibitory control and problem-solving) and academic performance in adolescents with depression. We focus both on inhibitory control [17] and planning (as an important aspect of problem-solving) as the latter seems to have particular relevance for self-study and preparation for exams in the adolescent population.

First, we aim to identify and analyse all studies in which inhibition and/or problem-solving (planning and/or decision-making) were assessed in depressed adolescents with MDD in comparison to age-matched controls. Second, we would like to analyse which neuropsychological tests were used to assess problem-solving and inhibitory control, as well as other aspects of executive functions, such as e.g. meta-tasking, in depressed adolescents.

## Methods

### Search strategy

The search was conducted in the Scopus database on 8 August 2022 with the use of the following search terms: ( TITLE-ABS-KEY (plan* OR multi-step OR problem-solving OR multi-task* OR *inhibition OR *shift* OR flexibility OR strategy OR decision) AND TITLE-ABS-KEY (adolescent* OR young OR youth OR teen*) AND TITLE (depress*) AND TITLE-ABS-KEY (*executive)) AND (LIMIT-TO (LANGUAGE, English")).

### Inclusion/exclusion criteria

The following inclusion and exclusion criteria were applied.

Inclusion criteria:

– Original studies with a healthy age-matched control group.
– Participants: 10–19 years (as defined by WHO 2023 [18] and/or mean age < 19.
– Diagnosis of depression (major depressive disorder) as defined by ICD-10, DSM-IV or DSM-V [19–21]
– At least 2 executive measures (either performance-based or clinician-rated) including at least one planning or decision-making measure.
– Texts in English.

Exclusion criteria:

– Diagnosis of bipolar affective disorder, schizophrenia or other psychotic disorder, neurodevelopmental disorders (e.g. autism), eating disorders.
– Drug/alcohol abuse.
– Other significant diseases (somatic diseases e.g. diabetes or cancer), neurological disorders (e.g. epilepsy, head trauma).
– Executive function assessment based only on self-ratings.

**Fig. 1:**
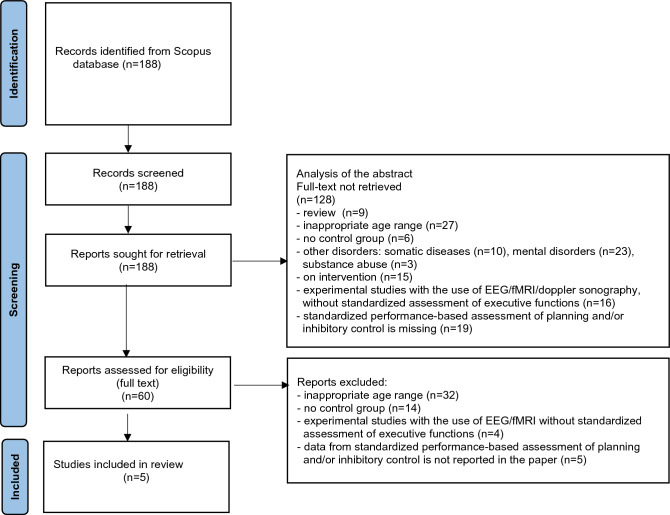
Flowchart detailing the literature search according to PRISMA methodology [27]

The search initially identified 188 documents (see: Fig. 1), amongst which 5 presented the results of studies which met the inclusion criteria for this review and did not meet the exclusion criteria. The studies are listed in Tables 1 and 2.

**Table 1:** Problem-solving and inhibitory control assessment in children and adolescents with major depressive disorder (MDD) in comparison to healthy controls (HC)

References and country	Participants (% males)	Age: mean age (SD) Age range (in MDD group if available)	Diagnostic criteria	Comorbid disorders	Exclusion criteria for MDD group	Medication	Problem-solving and /or inhibitory control tests	Measures	Results
Baune et al. [24], Australia	32 (21.9%) with MDD; 65 (46.1%) HC	MDD: 19.6 (± 2.9) 13–25	DSM-IV-TR	Not reported	Psychotic features; known neurological disorders; neurodevelopmental disorders such as learning disabilities affecting cognitive function; primary heavy alcohol and drug abuse	32 (41.9%), details not reported	*Planning:* Tower of *London Colorado Assessment Test (CATs)*	Excess moves for 3, 4 and 5 ring problems; average total trail time	No significant intergroup differences in any of the scores
Han et al. [22] USA	31 (38.7%) with MDD; 30 (46.7%) HC	MDD: 17.32 (± 1.59); 14.5–19.9 for the whole sample	DSM-IV	No comorbidity 3 (9.7%); any anxiety disorder 23 (74.2%) detailed comorbidities: Generalised Anxiety Disorder (48.4%); Attention Deficit Hyperactivity Disorder 4 (12.9%); Conduct Disorder (Childhood Onset) 2 (6.5%); Oppositional Defiant Disorder 4 (12.9%); Any externalizing disorder 7 (22.6%); Dysthymia 5 (16.1%); Generalised Anxiety Disorder 15 (48.4%); Panic Disorder 2 (6.5%); Obsessive–Compulsive Disorder 2 (6.5%); Posttraumatic Stress Disorder 7 (22.6%); Social Phobia 6 (19.4%)	An estimated full scale IQ < 80; significant medical or neurological disorders; a history of bipolar disorder, autism spectrum disorder, Schizophrenia, eating disorder within the past year, and/or substance-related disorder with a history of use in the past 60 days	Not medicated 11 (35.5%); Selective serotonin reuptake inhibitor (SSRI) 14 (45.2%) Atypical antidepressant 2 (6.5%) Tricyclic antidepressant (TCA) 1 (3.2%) Mood stabiliser 2 (6.5%) Atypical antipsychotic 1 (3.2%) Benzodiazepine 1 (3.2%) Stimulant 2 (6.5%) Norepinephrine reuptake inhibitor (NRI) 1 (3.2%) Serotonin-norepinephrine reuptake inhibitor (SNRI) 2 (6.5%)	*Affective decision-making*: the Iowa Gambling Task (IGT)	IGT (advantageous choices)	No significant inter-group differences
							*Inhibitory control:* the Attention Network Test (ANT),	ANT- alerting RT, orienting RT; conflict RT,	The conflict score reaction time was significantly higher in the MDD group
							*Inhibitory control:* the emotional Go-NoGo task;	Emotion Go/NoGo (fear, happy, calm)	No significant inter-group differences
							*Inhibitory control:* face No/NoGo task	Face No/NoGo (Go trials; nogo Trials)	No significant inter-group differences
Holler et al., [23] USA	22 (64%) with MDD; 22 (54%) with the minor depressive disorder; 33 (58%) HC	MDD: 15.70 (± 1.21); age range is not reported	DSM-IV	Anxiety- 45%; disruptive behaviour disorders—23%; ADHD- 36%	Bipolar disorder, a pervasive Developmental disorder Or a psychotic disorder	Not reported	*Planning:* The Rey- Osterrieth Complex Figure (ROCF) & WCST	The Rey-Osterrieth Complex Figure (ROCF)-unspecified score & WCST Categories;	MDD group scored lower than HC on the Executive Functioning Composite score; no significant inter-group differences in terms of planning or inhibitory control were noted
							*Inhibitory control:* Stroop test & WCST	Stroop Color Word (C-W) & WCST Failure to maintain set (FMS)	
Killey et. al. [25] Australia	38 (36.8% males) with MDD; 38 (52.6%) HC; 18 (50%) with first-episode psychosis	MDD: 20.41 (± 2.35); 16—25	DSM-V	Not reported	Not reported	Not reported	*Decision-making*: Mac Arthur Competence Assessment Tool-Treatment (MacCAT-T)	Understanding subscale; Appreciation subscale; Reasoning subscale; Expressing a choice subscale; MacCat-T Scale total,	Most participants within MDD group (84.2%) demonstrated adequate decisional capacity no difference in MacCAT-T between MDD and HC
Kyte et al. [26], UK	30 (40%) with MDD; 49 (40.8%) HC	MDD: 15.26 (± 2.50); age range is not reported	DSM- IV	Anxiety disorders(11); obsessive–compulsive disorder (7); substance misuse disorder (1); oppositional defiant disorder (5); conduct disorder (2); post-traumatic stress disorder (1); ADHD (1)	IQ below 70	Current antidepressant medication (3); a past history of SSRIs/SNRIs use (5)	*Decision-making:* The decision-making test	Speed of decision-making; quality of decision-making; risk adjustment	Longer decision time in HC no inter-group differences poorer risk adjustment in MDD
							*Inhibitory control:* The Affective Go, No-Go task	Response time; errors; ommissions	No inter-group differences MDD group performed better on sad words no inter-group differences

**Table 2:** Problem-solving and inhibitory control assessment methods in the context of multidimensional executive function assessment in children and adolescents with major depressive disorder (MDD )

Study	Initiation and maintenance	Planning and/or decision-making	Inhibitory control (monitoring/inhibition)	Working memory and/or attention (other than inhibitory)	Set-shifting/mental flexibility	Meta-tasking	Performance-based measures assessing other cognitive domains	Data on educational achievement	Comments
Baune, et al. [24], Autstralia	–	Tower of London from the Colorado Assessment Test (CAT)	–	n-back from CAT	Cartsort from CAT	–	verbal learning: task adapted from Rey Auditory Verbal Learning Test	Not reported	Groups not matched in terms of education level
Han et al. [22] USA	The Continuous Performance Task–Identical Pairs version (CPT-IP)	Affective decision-making: The Iowa Gambling Task (IGT)	The Attention Network Test (ANT); the emotional Go-NoGo task; face No/ NoGo task	The Continuous Performance Task–Identical Pairs version (CPT-IP)	–	–	Intelligence: the Wechsler Abbreviated Scale of Intelligence (WASI)	Not reported	MDD group scored lower on intelligence test than HC
Holler et al. [23], USA	Controlled Oral Word Association Test (COWAT) FAS and animals	The Rey–Ossterieth Complex Figure (ROCF) & WCST categories	Stroop CW & WCST FMS	WRAML sentence repetition and TMT A	Cognitive flexibility/set-shifting (TMT B & WCST perseverative errors)	–	Memory and learning: the Wide Range Assessment of Memory and Learning (WRAML)	Not reported	
Killey et. al. [25], Australia	–	Mac Arthur Competence Assessment Tool-Treatment (MacCAT-T)	–	TMT	–	–	Academic achievement: The Wide-Range Achievement Test-4 (WRAT-4) word reading subtest; verbal memory: the logical memory subtest (LM) from the Wechsler Memory Scale III	Not reported	Matching groups in terms of education is not reported; decisional capacity is unrelated to education level
Kyte et al. [26], UK	COWAT FAS and animals	The decision-making task	The affective go, no-go task	–	WCST; the intra-dimensional, extra-dimensional set-shifting task (ID-ED task from, Cambridge Neuropsychological Test Automated Battery (CANTAB)	–	Intelligence: Wechsler Intelligence Scale for Children-III	Not reported	

## Results

Our search identified 2 studies performed in the USA [22, 23], 2 studies from Australia [24, 25] and one UK study [26]. The number of participants in the MDD group ranged from 22 [23] to 38 [25], whilst the number of healthy controls ranged from 30 [22] to 65 [24]. Additionally, in one American study, there were 22 cases of minor depressive disorder [23], and in the newest Australian study, there were also 18 participants with first-episode psychosis [25]. The former study also used two control groups: healthy outpatients and inpatients hospitalised because of different reasons. The analysis of those results exceeds the scope of our review, so in Table 1, we report only data from the comparison of MDD patients with the outpatient group of HC.

In terms of age, the included studies covered a total age range from 13 to 25 years [24] or satisfied the appropriate mean age [22, 25]. In 2 papers, the participants’ age range was not explicitly reported [23, 26]. Of note, the clinical group was limited to adolescents in 3 studies [22, 23, 26] whilst in 2 remaining studies, young adults were also included [24, 25]. It is important as the development of frontal areas subserving executive functions continues till early adulthood [28]. However, as the age range is wide, as far as the adolescent population is concerned, quantitative scores could not be used for meta-analysis. As our search enabled us to identify only 5 papers, we decided to report the results of the studies that included also young adults.

The criteria used to diagnose MDD were not fully consistent across analysed studies. None of the studies used ICD-10 criteria. In 3 studies, DSM-IV criteria were applied [22, 23, 26], in one, DSM-IV-TR was used [24], and in the newest study, MDD was diagnosed in line with DSM-V [25]. However, the core clinical criteria for a depressive episode, as they are all derived from DSM classifications, are consistent both in terms of the number of symptoms required for a clinical diagnosis and the type of symptoms included. The only differences affect differential diagnosis with mixed episodes [19] or the impact of other states, such as bereavement [20, 21].

None of the studies reported quantitative educational achievement results (see: Table 2). In 2 studies, no data on the education level was provided [23, 26]. Healthy controls were not matched to MDD participants in terms of intelligence. In one of the studies, the clinical group had significantly lower intelligence than healthy controls (HC) [22]. In one study, IQ below 70 [26] or an estimate of IQ < 80 [22] was used as an exclusion criterion.

In one of the papers, exclusion criteria were not provided at all [25]. In terms of comorbidities reported as exclusionary, the following disorders are mentioned: bipolar disorder in 2 studies [22, 23], psychotic disorder and/or schizophrenia in 3 studies [22–24]; current or recent substance use in two studies [22, 24], eating disorder in 1 study [22], autism spectrum disorder in 1 study [22], a (neuro)developmental disorder in 1 study [24] and known neurological and/or significant medical disorder in 2 studies [22, 24]. As far as comorbidities reported in MDD groups are concerned, anxiety disorders were the most common [22, 23, 26]. Cases with comorbid attention deficit with hyperactivity disorder (ADHD) were included in 3 studies [22, 23, 26]. Of note, two studies did not describe any comorbidities in the MDD group [24, 25]. Interestingly, none of the studies reported information on non-pharmacological interventions such as psychotherapy. Medication was described in only 2 studies [22, 26]. The most common pharmacological treatment was antidepressant medication (SSRI). In one of the studies [22], the patients received stimulants, which may influence executive function [29].

### Methodology of executive function assessment

As depicted in Table 2, none of the studies assessed all aspects of executive functions. However, 4 out of 5 studies used more than 2 executive tests [22–26]. Of note, none of the studies reported an assessment of meta-tasking, which seems a very important ecologically valid aspect of executive assessment [5].

### Problem-solving assessment

The methods of planning and decision-making assessment were very heterogeneous. Planning was assessed in only 2 studies [23, 24], and only in one of them, standardised scoring is reported. Baune et al. [24] used one of the most popular tests to assess planning—Tower of London (TOL) [24]. In the second study, reporting planning assessment only a composite score is described, encompassing data from Rey–Osterrieth Complex Figure Test (ROCF) and Wisconsin Card Sorting Test score (WCST Categories completed) [23]. In terms of decision-making, three different tasks were used: Mac Cat [25], The Decision-Making Task [26] and The Iowa Gambling Task (IGT) [22]. The last study is the only one which used a decision-making task engaging hot executive functions.

### Inhibitory control assessment

Inhibitory control was assessed in 3 studies and each of them used a different methodology. Han et al. [22] used 2 tasks ANT, The emotional go/no-go task to address inhibition [22]. Holler reported only a composite score based on Stroop Color-Word and WCST Failure to Maintain Set (WCST FMS) [23] whilst Kyte et al. used only one experimental affective go/no-go task [26].

### Problem-solving in adolescents with MDD

No differences between adolescents with MDD and HC were shown in terms of planning in any of the 2 studies, regardless of methodology [23, 24]. As far as the quality of the decision-making is concerned, a clear-cut deficit was not identified either by Killey et al. [25] or Kyte et al. [26]. Whilst poor risk adjustment was evidenced by Kyte et al. in The Decision-Making Task, it was not shown in the IGT [26].

### Inhibitory control in adolescents with MDD

The pattern of results in terms of inhibitory control is inconsistent across studies. In the study by Han et al. [22], only 1 out of 3 inhibitory control tests evidenced worse performance in individuals with MDD. Holler et al. [23] and Kyte et al. [26] did not document deficient performance in the MDD group and only qualitative differences were present in an experimental task requiring emotional processing.

### Academic performance and intelligence

In only one study, academic achievement was assessed with The Wide-Range Achievement Test-4 (WRAT-4) word reading subtest [25]. In the same study, decisional capacity was unrelated to education level. Intelligence tests were used in only two studies, either in full format [26] or a shortened one to provide an estimate of IQ [22]. The latter study group with MDD scored lower on the intelligence test.

### Academic performance in relation to selected executive processes in adolescents with MDD

Educational attainment in terms of exam results or final grades is not reported in any of the reviewed studies. In the Australian studies, groups were either not matched in terms of education level [24] or data on matching the groups is not reported [25].

Summing up, the relationship between educational attainment and problem-solving and inhibitory control in adolescents with MDD cannot be established on the basis of the existing literature.

## Discussion

We conducted a systematic review of EF in terms of problem-solving and inhibitory control in adolescents with MDD in the context of educational achievement. We wanted to narrow our literature analysis to the adolescent subgroup, allowing studies with young adults but not with young children, because of two reasons. First, frontal structural connectivity, underpinning executive functions, develops dynamically between adolescence and early adulthood [30]. Second, as educational attainment in adolescence may rely more on the effective performance of multi-step goal-directed activities and not only on inhibition, we aimed to focus on planning, decision-making and meta-tasking. Surprisingly, the topic has not received sufficient attention in the literature.

The applied search strategy identified only 5 studies, published between 2005 and 2022. Many studies were excluded as they did not focus on adolescents but on younger children. Very few studies included the adolescent population exclusively, most often this age group was mixed either with children or young adults.

Unfortunately, none of the studies reported data on educational attainment which, from our perspective, seem to be particularly significant information. This problem requires further study as in adolescence school performance is highly important. What’s more, this lack of data on the effect of depression in adolescents on school performance contrasts with the literature on work performance in adult depression. The severity of depression in adults is related to a decrement in work performance and symptom relief does not fully eliminate the adverse work outcome of depression [31]. In children aged 8–12 years, depressive symptoms are associated with poorer school performance [32]. In medical students, fewer depressive symptoms are associated with higher college grade-point [33]. As far as the adolescent population is concerned, in those aged 14–18, lower grades are related to more depressive symptoms [34, 35]. Also, self-reported depression symptoms in 7th grade are associated with the increased risk of dropping out of school in later adolescence [36].

It was previously hypothesised in adults that executive performance could moderate the effect of depression on work performance [37]. The same effect could be present in adolescents with regard to school performance. Albeit this hypothesis was not confirmed in the adults using self-report executive measures [37], it is worth verifying in a study including data on educational attainment and performance-based comprehensive measures of executive function in the adolescent population.

In most of the papers that we analysed, as well as in those previously reviewed by Vilgis et al. [4], most of the studies focussed on inhibitory control, whilst very few addressed planning or decision-making. Overall, the pattern of the results is similar to the one reported by Vilgis et al. [4].

As in studies reviewed by Vilgis et al. [4], results on planning were inconsistent. Whilst deficient planning was not evidenced in one study [24], in another one, the results, due to a composite planning/problem-solving [23] (based partially on scores from the Wisconsin Card Sorting Test that is a test of set-shifting), were inconclusive. Also, no statistically significant differences were observed in terms of planning/problem-solving between individuals with MDD and those with minor depression [23].

Vilgis et al. [4] identified 3 studies addressing planning, in 2 of which deficits were ascertained. As far as decision-making is concerned, deficits were revealed only in about 15% of depressed patients in one of the studies [25] but not in the two remaining studies [22, 26]. Of note, decisional capacity in adolescents with MDD was better preserved than in adolescents with first-episode psychosis, as in the latter group problems were observed in 66% of cases.

Inhibitory control assessment also yielded negative results in three studies [22, 23, 26], whilst amongst all studies reviewed by Vilgis et al. [4], only 3 out of 16 studies provided positive results. As two of those previously reviewed positive studies included children [38, 39] whilst our review focussed exclusively on adolescents, inhibitory control deficits may be more likely to present at an earlier age and are not a typical feature of depression in adolescence. This speculation would require confirmation in further research. Of note, inhibitory control was comparable in adolescents with MDD and those with minor depression [23]. It is unclear if negative results in terms of inhibitory control are conclusive or not, as in each of the studies different task was used. Two of them used the go/no-go paradigm, whilst in one of them, Stroop interference task was used. Also, one of them used an emotional (hot) task, whilst the other used cognitive (cold) tasks. The neural correlates inhibitory dimensions may dissociate, as was elegantly shown by a meta-analysis of 66 study experiments including 1447 participants [40]. The only common area engaged in inhibitory processes overall is the left anterior insula, whilst cognitive inhibition is related to the dorsal frontal inhibitory system and emotional interference engages a ventral inhibitory system. Till now, there is no evidence to confirm either generalised or specific inhibitory deficits in depressed adolescents. However, it seems that cognitive inhibition is particularly worth studying in the context of educational achievement.

Overall, planning and decision-making are not commonly assessed in depressed adolescents, even in studies focussing on executive function. Both the existence and the possible severity of problems in these areas require further study.

As comorbidities were not reported in all analysed studies, the effect of them on executive functioning in depressed adolescents cannot be fully determined. Anxiety disorder and ADHD were amongst the most common comorbid disorders in the studied adolescent populations with depression. Only in one study, the co-occurrence of ADHD was used in the statistical analysis. Holler et al. [23] showed that using behavioural disorder and ADHD diagnoses as covariates did not modify the pattern of results. Of note, inattention as a symptom of ADHD may predict the later development of depression in children [41].

As depression is frequently accompanied by anxiety disorders, further research is needed to fully understand the relevance of rumination, coping strategies, motivation and locus of control, self-efficacy, personality traits (e.g. perfectionism) and psychosocial variables for academic performance in adolescents with depression. Interestingly, previous research indicated that in early adolescence, rumination affects sustained attention. Rumination predicted better-sustained attention in those with low levels of depressive symptoms and worse-sustained attention in those with high levels of depressive symptoms [42]. Depressive symptoms are related to avoidant coping strategies in young adults and depressive symptoms remain the main predictor of coping strategies in this cohort [43]. It can be hypothesised that the same association is present in younger individuals.

The relationship between perfectionism, as a personality trait, depressive symptoms and academic achievement seems worth further study in the context of executive skills. Stoeber & Rambow [44] showed that whilst striving for perfection is related to academic success in adolescent school students and negative reactions to imperfections are likely to undermine motivation. Of note, when the effect of negative reactions was controlled for, perfectionism was negatively related to depressive symptoms. It would be interesting to find out, if executive function, especially set-shifting could moderate these relationships.

When it comes to self-efficacy in depressed adolescents, self-efficacy is inversely related to depression as a general phenomenon [35, 45], but this relationship was not confirmed for academic self-efficacy [45].

Also, it was shown that adult attachment style in students is an important factor modulating academic performance [46]. It can be hypothesised that this factor may be of even more importance at the earlier stages of development—e.g. early adolescence.

Summing up, it seems that a comprehensive study on the relationship between executive function and educational achievement should encompass not only the performance-based measures of very well-defined executive skills but also questionnaires and structured/semi-structured interviews focussing on coping strategies, motivation and self-efficacy and perfectionism, as they are also likely to be related to educational success, especially when long-term engagement in exam preparation is required.

## Conclusion

The review identified a considerable gap in the research on the functional impact of depression in adolescents. The existing literature does not allow us to confirm the relationship between executive functioning and academic performance in adolescents with MDD. Further studies, focussing on planning, decision-making and meta-tasking, including data on educational attainment and psychosocial variables in adolescents with MDD are needed. Identifying the link between specific executive deficits, personality traits and coping strategies and school performance could guide tailored therapeutic interventions. By recognising this problem at an early stage of diagnosis, clinicians could modify their interventions/protocols, paying more attention to the, so far, unrecognised area of functioning of adolescents diagnosed with depression.
